# Supplementary material for: In Vivo and In Vitro Cartilage Differentiation from Embryonic Epicardial Progenitor Cells
Source: Int J Mol Sci. 2022 Mar 25;23(7):3614. doi: 10.3390/ijms23073614 (PMC8999123; doi:10.3390/ijms23073614)
Supplement: Supplementary file 1 [file ijms-23-03614-s001.zip › ijms-1612790-supplementary.pdf]

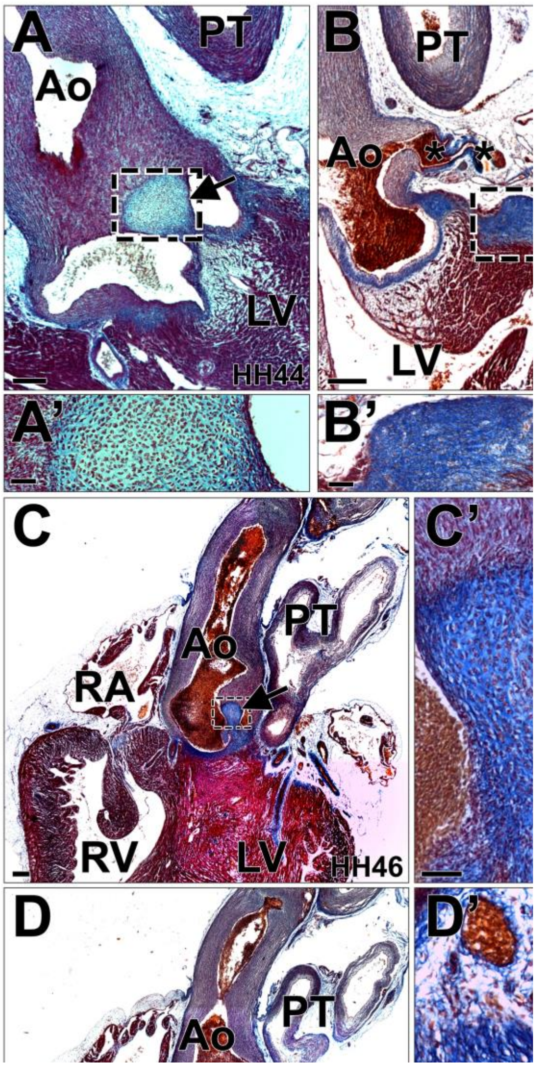

Figure S1. Cartilage clusters in the embryonic chick heart. (A-C') Mallory's trichrome staining of chick heart sections (perinatal stages). Cartilage clusters are shown in the aorta of HH44 (A, A') and HH46 (C,C') embryos. Additional cumuli of fibrous-connective tissue are observed in the left atrioventricular sulcus of HH 44 (B, B') and HH46 (D, D') chick embryos. Asterisks point to the left coronary aortic sinus (*, B). Abbreviations: Ao, aorta; LA, left atrium; LV, left ventricle; PT, pulmonary trunk; RA, right atrium; RV, right ventricle. Scale bars = A,B,C,D: 200µm; A',B',C',D': 50µm.

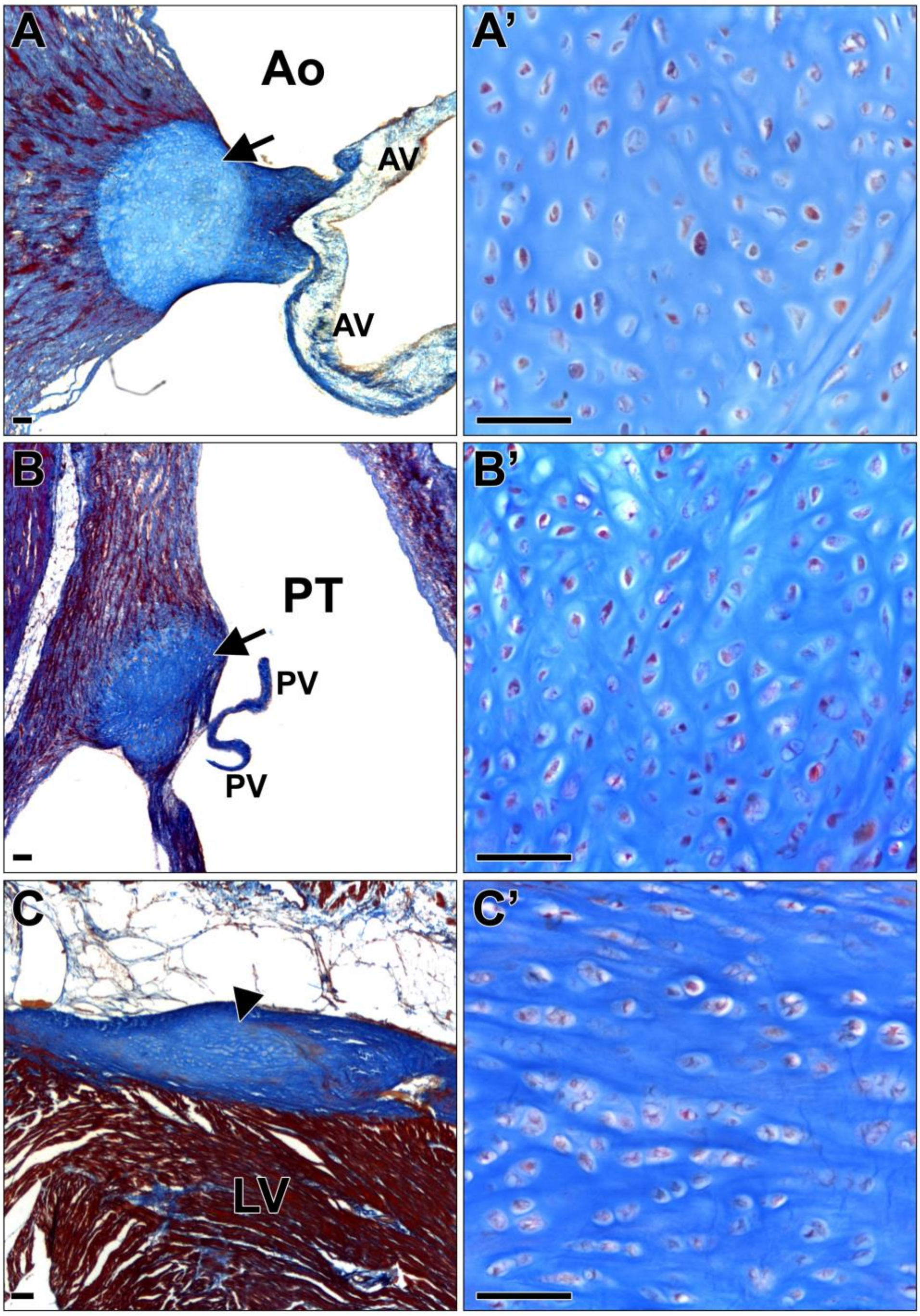

Figure S2. Cartilage clusters in the adult chick heart. (A-C') Mallory's trichrome staining of adult chick heart sections. Cartilage is shown in the aorta (A, arrow; magnified in A'), pulmonary trunk (B, arrow; magnified in B') and left atrioventricular sulcus (C, arrowhead; magnified in C'). Abbreviations: Ao, aorta; AV, aortic valve; LV, left ventricle; PT, pulmonary trunk; PV, pulmonary valve. Scale bars = A,B,C: 100μm; A',B',C': 50μm.

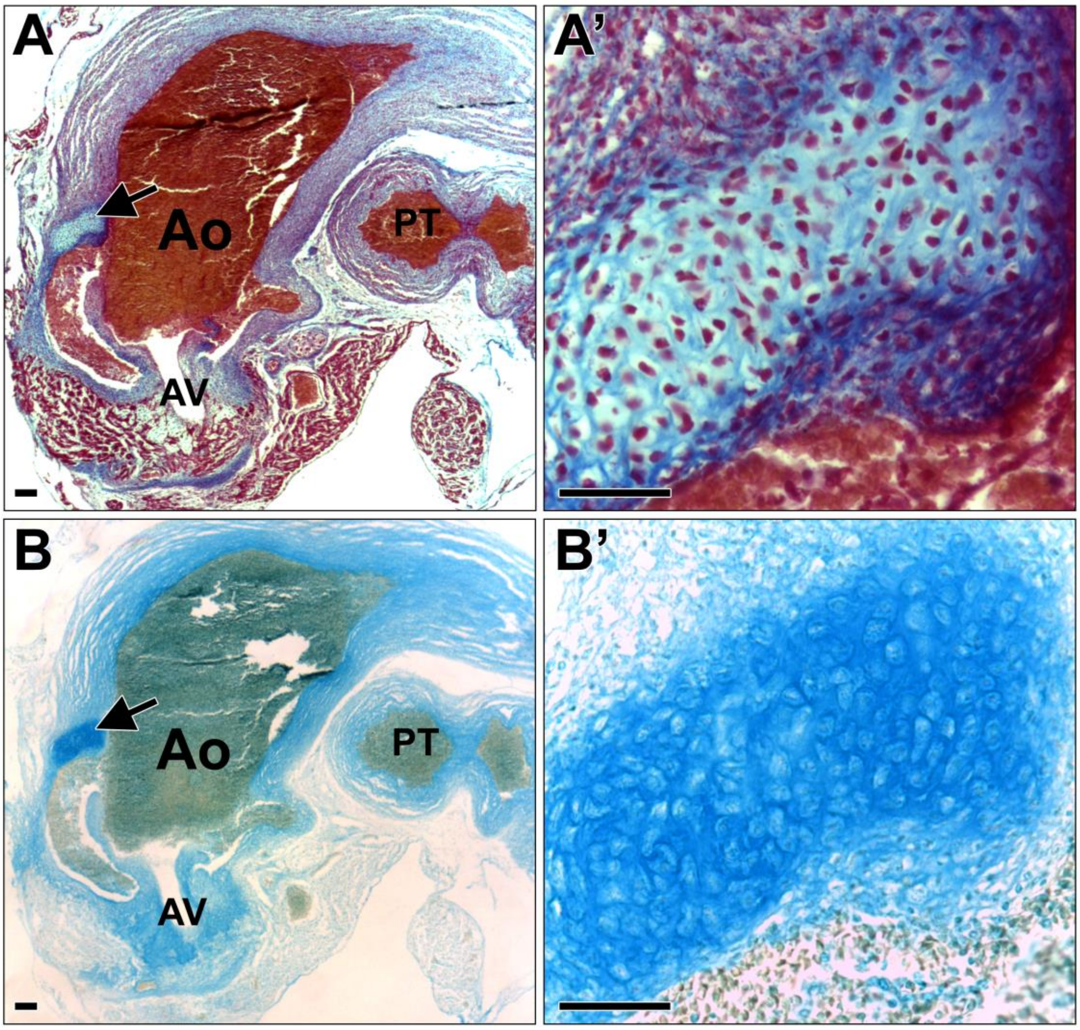

Figure S3. Normal anatomy of the quail aortic root. (A,A’) Mallory trichrome staining of the aortic root of a quail embryo (HH44), showing the aorta (Ao) and pulmonary trunk (PT). (B,B’) Sections from the same heart, Alcian blue staining. Arrows point to a cartilage cluster in the aorta (Ao, A, B; magnified in A’ and B’, respectively). Abbreviations: Ao, aorta; AV, aortic valve; PT, pulmonary trunk. Scale bars: A,B= 100µm, A’,B’ = 50µm.

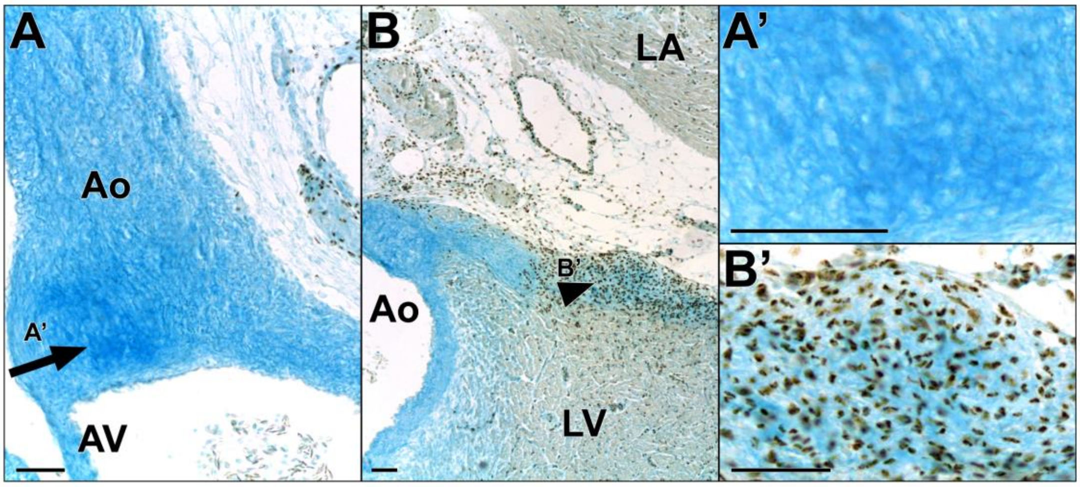

Figure S4. Proepicardial derived cells differentially differentiate into cartilage in the avian ventricle, but not in the cardiac outflow tract. (A-B') Alcian blue and QCPN co-staining (black nuclei indicate QCPN-positive cells) of a quail to chick PE chimera (HH44), revealing the absence of proepicardial derived cells in the aorta (A). The staining highlights the aortic cartilage (A, arrow; A'), and the presence of proepicardial derived cartilage in the left atrioventricular sulcus (LOT) (B, arrowhead; B'). Abbreviations: Ao, aorta; AV, aortic valve; LA, left atrium; LV, left ventricle. Scale bars = 50µm.

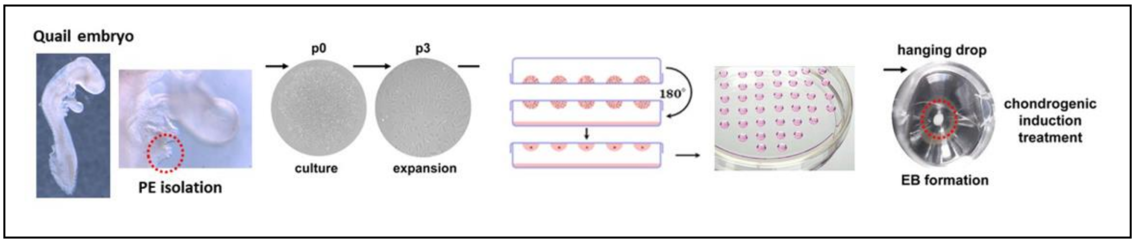

Figure S5. Chondrogenic differentiation protocol for cultured quail PE. Major steps during *in vitro* differentiation of proepicardial cells include the isolation of PE from quail embryos, the expansion of these cells in vitro (up to the third passage), the formation of embryoid bodies in hanging drop cultures, and the incubation of cells in a chondrogenic medium (21 days). Abbreviations: p, passage; PE, proepicardium.
